# RMDAP: A Versatile, Ready-To-Use Toolbox for Multigene Genetic Transformation

**DOI:** 10.1371/journal.pone.0019883

**Published:** 2011-05-13

**Authors:** Lei Ma, Jiangli Dong, Yongsheng Jin, Mingliang Chen, Xiaoye Shen, Tao Wang

**Affiliations:** State Key Laboratory of Agrobiotechnology, College of Biological Sciences, China Agricultural University, Beijing, China; Cairo University, Egypt

## Abstract

**Background:**

The use of transgenes to improve complex traits in crops has challenged current genetic transformation technology for multigene transfer. Therefore, a multigene transformation strategy for use in plant molecular biology and plant genetic breeding is thus needed.

**Methodology/Principal Findings:**

Here we describe a versatile, ready-to-use multigene genetic transformation method, named the Recombination-assisted Multifunctional DNA Assembly Platform (RMDAP), which combines many of the useful features of existing plant transformation systems. This platform incorporates three widely-used recombination systems, namely, Gateway technology, in vivo Cre/*lox*P and recombineering into a highly efficient and reliable approach for gene assembly. RMDAP proposes a strategy for gene stacking and contains a wide range of flexible, modular vectors offering a series of functionally validated genetic elements to manipulate transgene overexpression or gene silencing involved in a metabolic pathway. In particular, the ability to construct a multigene marker-free vector is another attractive feature. The built-in flexibility of original vectors has greatly increased the expansibility and applicability of the system. A proof-of-principle experiment was confirmed by successfully transferring several heterologous genes into the plant genome.

**Conclusions/Significance:**

This platform is a ready-to-use toolbox for full exploitation of the potential for coordinate regulation of metabolic pathways and molecular breeding, and will eventually achieve the aim of what we call “one-stop breeding.”

## Introduction

The vast majority of conventional transgenic plants are produced to improve agronomic traits, through genetic transformation of only one transgenic protein. However, in organisms, traits and physiological functions rely on coordination of multigene expression. Therefore a strategy for simultaneous transfer of multiple genes into plants will become the future of genetic engineering technology and provide the potential for manipulating sophisticated metabolic pathways, expressing recombinant protein complexes, and studying complex genetic control circuits for crop improvement [1].

*Agrobacterium* vectors have been developed, extensively improved and modified to perform various user-friendly applications, and are currently the favored DNA delivery method for plant genetic transformation [2], [3]. Although these vectors are operable in plant science and biotechnology, their ability to simultaneously express several target genes from a single plasmid is limited by their basic design [4]. A number of strategies, have been employed to introduce multiple genes into plant genomes [4], [5]. Generally, the application of stacking multicassettes into one T-DNA has an advantage over the transformation methods of single-gene vector, in that the former would minimize complex integration patterns [4], and reduce the transformation steps needed to engineer the desired genotype. Traditional cloning methods have proved feasible in the assembly of large binary plasmids [6], but are limited in that they lack unique restriction sites during iterative cloning: the more exogenous genes are stacked, the less restriction sites can be used. Homologous recombination and site-specific recombination are widespread genetic phenomena in nature. Based on these two systems, various gene manipulation methods have been created and widely used. Homologous recombination is common in *Escherichia. coli*, *Saccharomyces cerevisiae*, and several other species. Two highly engineered cloning methods of mating-assisted genetically integrated cloning (MAGIC) *in vivo* [7], together with sequence and ligation–independent cloning (SLIC) *in vitro* [8], have all been developed to facilitate the construction of recombinant DNA molecules. Yeast recombination has since been applied to construct plasmids and yeast artificial chromosomes (YACs), and is considered an ideal tool for sequence-specific assembly of plasmids or even genomes [9], [10]. So far, these methods still stay at the level of microorganisms; it will be some time before these can be used in plant genetic breeding. Furthermore, a system combining the Cre/*lox*P recombination system and two rare-cutter endonucleases was reported for the sequential assembly of multiple genes onto a transformation-competent artificial chromosome (TAC) vector [11]. MultiSite Gateway technology [12] and MultiRound Gateway technology [13], based on Gateway cloning to deliver multiple transgenes into a destination vector, were subsequently developed. Meanwhile, Iterative Site Specific Integration (ISSI) [14] and Multiple Round *In Vivo* Site Specific Assembly (MISSA) [15] for DNA assembly were also developed.

As mentioned above, a number of improved methodologies have been reported for multigene transformation, but the vast majority of existing systems only focus on how to stack DNA fragments. In general, the users were provided with empty plasmids lacking *cis*-regulatory elements (promoters, terminators, or other genetic elements) of a gene of interest [16], and few systems are available directly for plant molecular genetics, especially for crop improvement. We combined the advantages of existing genetic transformation systems aiming to achieve what we call a “one-stop Breeding” system, i.e., a system that can easily provide the requirements for vector construction in genetic engineering. The Recombination-assisted Multifunctional DNA Assembly Platform (RMDAP) described here currently has 14 pairs of satellite vectors (pOSB series) and three kinds of recipient vectors, designed for the needs of transgenic research (e.g., overexpression, RNAi and marker-free vectors), with the aim of resolving practical problems of plant genetic improvement. In addition, Gateway technology, *in vivo* Cre/*lox*P, and recombineering recombination systems were employed to assemble DNA fragments, ensuring high efficiency and accuracy. This is a versatile, easy-to-use multigene genetic transformation platform for the assembly of multiple-gene expression cassettes to meet various requirements of plant genetic breeding.

## Results

### Methods of DNA assembly

RMDAP contains donor and recipient vectors, and uses bacterial strain SW106 as the recipient host, which has all the requisite *in vivo* recombination proteins, including an arabinose-inducible *cre* gene and a defective *λ* prophage with recombination proteins *exo*, *bet*, and *gam* controlled by the temperature-sensitive repressor *cI*857 [17]. The assembly of DNA fragments was carried out by Cre/*lox*P-mediated and Red-mediated recombination events alternately and circularly.

**Figure 1:**
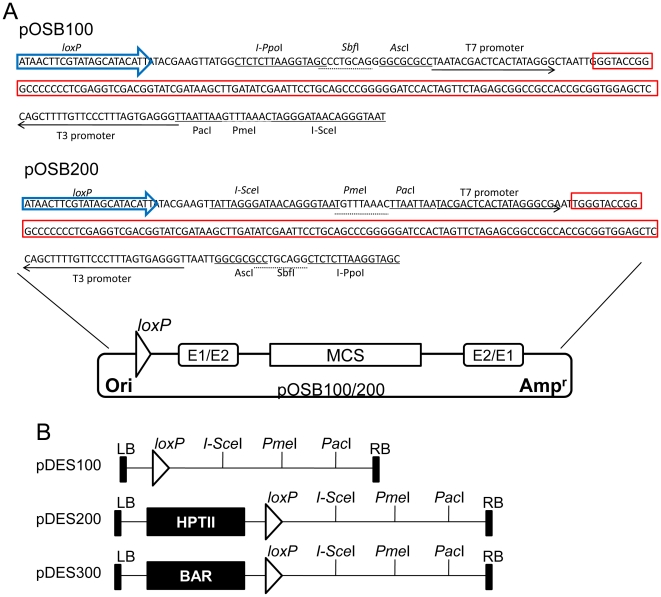
Physical maps of original donor vectors and recipient vectors carrying different marker genes. (A) Schematic illustration of the two original entry vectors pOSB100 and pOSB200, from which the series is derived. The fragment introduced into the pMD18-T simple vector contained a *lox*P site (blue arrow), a multicloning site (red box), and two rare restriction enzyme sets E1/E2 (underlined). These two sets are in opposite orientation. Ori, pUC replicon; Amp**^r^**, ampicillin resistance gene. (B) Construction of binary transformation vectors equipped with no plant selection gene, for marker-free type assembly, or the commonly used plant selection genes of *hptII* and *bar*, based on the pCAMBIA family. LB and RB, left border and right border of the T-DNA; HPTII, hygromycin resistance gene cassette; BAR, phosphinothricin resistance gene cassette.

First, two original donor vectors, pOSB100 and pOSB200, were constructed (Figure 1A) containing the sequences “*lox*P+E1+MCS+E2” and “*lox*P+E2+MCS+E1” respectively (E1 = rare restriction enzyme set 1, MCS = multiple cloning site, E2 = rare restriction enzyme set 2). We chose the pMD18-T simple vector (Takara) as a skeleton with ampicillin resistance, and MCS from p*Bluescript* SKII. Rare restriction enzyme set 1 (E1) contains *Asc*I, *Sbf*I, and homing endonuclease *I-Ppo*I sites, while rare restriction enzyme set 2 (E2) contains *Pac*I, *Pme*I, and homing endonuclease *I-Sce*I sites. The homing endonucleases are double-stranded DNases with large, asymmetric recognition sites (12–40 bp) (New England BioLabs), so recognition sites are extremely rare. As *Pac*I, *Pme*I, *Asc*I, and *Sbf*I are the restriction enzymes able to identify eight nucleotide sequences, the occurrence of these is also relatively low, and so they could been carried out identification of the vector by enzyme digestion, and if necessary, also excise the intermediate vector's backbone. All satellite vectors later described are derived from these two original vectors. The RMDAP inherits all of the advantages of the former system [11], [16], but also has greatly expanded functional and utilization aspects. These methods are completely compatible.

Based on the backbone of vector pCAMBIA (CAMBIA, Canberra, ACT, Australia), we designed and constructed a set of three T-DNA destination vectors for various tasks, carrying no plant selectable marker gene or genes encoding resistance to hygromycin (*hpt*II, hygromycin phosphotransferase) and phosphinothricin (*bar*, phosphoinothricin acetyltransferase). The component containing *lox*P and E1 was cloned and introduced into corresponding sites of pCambia1300 and pCambia3301, producing pDES100, pDES200 (HPTII), and pDES300 (BAR) (Figure 1B). In particular, destination vector pDES100 was used to construct a maker-free binary vector, combined with satellite plasmid containing a plant selection gene that could be induced to allow removal.

**Figure 2:**
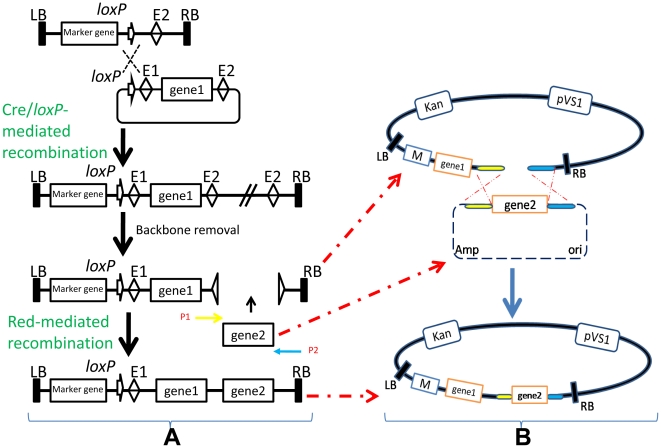
Schematic diagram for the round of gene stacking of the first two genes. (A) Through *in vivo* Cre/*lox*P-mediated recombination, the donor plasmid with the first gene was integrated into the recipient vector via *lox*P. Then the backbone of donor plasmid was excised to form a linear targeting construct. Next, the second gene or DNA fragment was retrieved by *in vivo* Red-mediated recombination and gap repair. (B) Detailed schematic illustration of how Red-mediated recombination might occur. E1/2, rare restriction enzyme set 1 / 2; Ori, pUC replicon; Amp**^r^**, ampicillin resistance gene; Kan, kanamycin selection gene; pVS1, pVS1 replicon; M, marker gene; LB and RB, left border and right border of the T-DNA.

Before assembling the DNA fragments, according to the specific purpose of the experiment, we need to choose appropriate satellite vectors. The assembly of the first two genes is shown schematically in Figure 2A. We adopted *in vivo* Cre/*lox*P-mediated recombination via *lox*P to allow the donor vector pOSB1xx to co-integrate with the recipient vector. A mixture of donor vector and recipient vector was electroporated together into *E. coli* strain SW106, which had been induced expression of the *cre* gene by adding filter-sterilized arabinose. This formed a vector mixture containing the co-integrated vector and parent vectors in the same strain. To create pure clonal populations of the recombinant, it was necessary to retransform it to *E. coli* DH5α at a concentration of less than one molecule per cell. The resulting co-integrative plasmids were mini-preped and purified to be digested completely by the homing endonuclease to remove the backbone. Next (Figure 2B), we performed Red-mediated recombination to retrieve another fragment. A pair of primers, each with at least 50 base homologies at the 5′-ends corresponding to the flanking sequence of the homing endonuclease site, and about 20 bases of the second gene or functional fragment were designed to amplify a target DNA fragment through high-fidelity Taq DNA polymerase. The resulting gene segment was ligated to the TA-cloning vector, followed by sequencing. The correct plasmid was transformed into strain SW106 to prepare competent cells after induction of the *λ* recombination genes. Finally, the linear vector fragment excluding the skeleton of the donor plasmid was transformed into the SW106 cells to achieve recombineering, and then screened for the correct clone on the right medium. At this point, two genes of the first round were integrated into the expression vector. For the second round of gene integrations, we chose pOSB2xx as the donor vector. In short, heterologous fragments could been stacked into the expression vector alternately according to the above procedures, in only a few days, using simple molecular biology operations.

### Features of the modular vector

Functional analysis for crop improvement can be approached in a number of ways, most of which rely on overexpression of transgenes, RNAi of endogenous genes to manipulate biological processes in transgenic plants, and marker-free vectors, related to the safety of genetically modified organisms (GMO). Therefore, the specific design of the satellite vectors is based on these three aspects: overexpression vector with different promoters, RNA interference vector, and marker-free vector.

**Figure 3:**
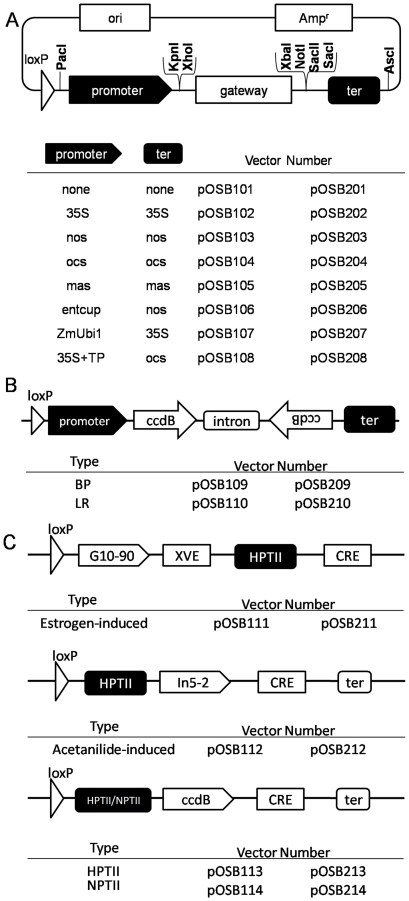
Schematic representations of the pOSB modular vector series. There are three functional types of modular vector containing 14 pairs of satellite vectors. In addition to special illustrations, pOSB2xx vectors can be digested using restriction enzymes *Pac*I (*Pme*I) and *Asc*I (*Sbf*i) to obtain the whole expression cassette, and inserted into pOSB100, resulting in pOSB1xx. (A) The gateway destination cassettes consist of R1 (attR1 recombination attachment site), ccdB (negative selection marker), and R2 (attR2 recombination attachment site) sequences, flanked by *Pac*I, *Kpn*I, *Xho*I and *Xba*I, *Not*I, *Sac*II, *Sac*I, allowing the replacement with different regulatory elements. All promoters and terminators (cauliflower mosaic virus 35S promoter(p*35S*), nopaline synthase gene promoter (p*NOS*), octopine synthase promoter (p*OCS*), manopine synthase promoter (p*MAS*), tobacco cryptic promoter (p*ENTCUP2*), and maize ubiquitin promoter (p*UBI*)) are introduced into corresponding sites, producing a variety of constitutive ectopic gene expression vectors. The chloroplast-targeted tissue-specific expression vector was constructed through the ribulose 1,5-bisphosphate carboxylase small subunit of tobacco driven by promoter p*35S*. (B) Outline of the different RNAi vectors produced in this study. Two inverted repeats of the gateway destination cassettes containing attP or attR sites locate on both sides of the intron driven by promoter p*CaMV35S* promoter tetramer. (C) Three types of marker-free satellite vectors: an estrogen-inducible marker-free vector, an acetanilide -inducible marker-free vector and a general-purpose type of marker-free vector. Ori, pUC replicon; Amp**^r^**, ampicillin resistance gene; ter, terminator; HPTII, hygromycin resistance gene cassette; NPTII, kanamycin resistance gene cassette; G10-90, a strong synthetic constitutive promoter; XVE, a chimeric transactivator containing the regulatory domain of an estrogen receptor; CRE, Cre recombinase with an intron.

Gain-of-function can be achieved by placing a gene of interest under the transcriptional control of a constitutive promoter. When carrying out multigene transformations, adoption of the homologous promoter has been found to creat certain transgene-silencing effect [18], making different promoters of the expression cassette necessary. Thus, to design modular vectors for constitutive ectopic expression, we developed a set of six couples of modular plasmids (Figure 3A), each of which carried a different pair of promoter and terminator sequences: cauliflower mosaic virus 35S promoter (p*35S*), nopaline synthase gene promoter (p*NOS*), octopine synthase promoter (p*OCS*), manopine synthase promoter (p*MAS*), tobacco cryptic promoter (p*ENTCUP2*) strong, constitutive promoters constructed for dicot plants, and maize ubiquitin promoter (p*UBI*) for monocot plant transformation. Besides these, another chloroplast-targeted satellite vector was also constructed successfully (Figure S1). Meanwhile, because the production of the constructs is laborious and often hampered by inappropriately positioned restriction sites, we also adopted Gateway cloning technology to join fragments in a predefined order, orientation, and reading frame [19]. Due to the presence of accessible restriction sites, we can further extend the family of plasmids to carry various promoters and terminators, especially inducible or tissue-specific promoters, through traditional ligation to maintain the high flexibility of the system.

Long double-stranded RNAs, in particular hairpin RNAs (hpRNAs), can be used to trigger gene silencing by RNA interference, and have been used for highly effective post-transcriptional gene silencing in plant cell research [20]. Using vectors of p*Hellsgate* proved to be a straightforward method for assembly by Gateway recombination of hpRNA constructs, designed for high-throughput gene silencing in plant cells [21]. Through the LR or BP reaction with *att*L1(P1)-trigger-*att*L2(P2), the trigger sequence is incorporated into both sides of the intron in opposite orientations. For the RNA-interfering modular satellite vector, we adopted this series of vectors with the *Not*I restriction site and succeeded in transferring the RNAi expression cassette to the intermediate vector (Figure 3B). Because the p*Hellsgate* vectors allow production of an hpRNA construct in a one-step or two-step process, we also retained these two ways.

Although no reports describe the effects of selectable marker genes on human health or environmental safety, public concerns over the safety of transgenic plants cause fierce debate. Selectable marker gene facilitates genetic transformation of plants, but generally it is not required for expression of the trait gene in the transgenic plant, and may even be harmful. How to delete a selectable marker gene has become an important research topic in the application of transgenic research. The Cre/*lox*P recombination system of the *E. coli* bacteriophage is a commonly used technique, and we took advantage of the general applicability of this system in bacteria and plants, i.e., the assembly of several transgenes in bacteria and removal of the selectable marker gene in plants by the inducible promoter, to construct two chemically induced marker-free satellite vectors. When foreign genes are introduced into plants, it is possible to remove the marker gene *in vivo* using estrogen [22] or acetanilide [23]. Meanwhile, to meet the needs of genetic breeding, we also generate a general-purpose type of marker-free vector in which the selection marker genes *hpt*II and *npt*II (neomycin phosphotransferase II) were preset. By virtue of Gateway recombination, we can rapidly replace any inducible or tissue-specific promoter (Figure 3C).

### Construction of plant multigene transformation vectors

As proof-of-principle experiments to evaluate the application of this modular vector system for multigene delivery, two transformation vectors with different expression units for different aims of genetic improvement were developed. Several genes, including *Atriplex hortensis* betaine aldehyde dehydrogenase (*badh*) involved in the accumulation of glycine betaine giving tolerance to salt stress [24], isoflavone synthase (*ifs*) from *Medicago truncatula* that a key enzyme in the flavonoids/isoflavonoids pathway [25], a phosphinothricin resistance gene (*bar*) and two reporter genes, *gus* and *gfp*, were chosen for gene stacking.

**Figure 4:**
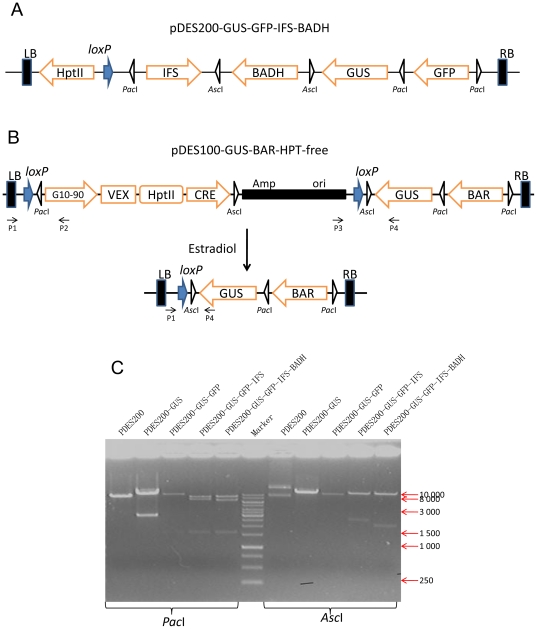
Schematic representation of plant transformation vector. (A) Schematic structures of plant transformation vector pDES200-GUS-GFP-IFS-BADH. The T-DNA with left and right borders (LB and RB) is indicated. The different expression units of IFS cassette, BADH cassette, GUS cassette and GFP cassette, including the HPTII cassette, are indicated by orange arrows. (B) The inducible marker-free multigene transformation vector pDES100-GUS-BAR-HPT-free contains two interested genes of *gus* and *bar*. The *hptII* gene is responsible for hygromycin resistance needed for transgenic event selection and flanked by two direct repeat *lox*P sites, which could be excised by the induction of estrogen. (C) Restriction analysis of recombinant constructs containing different numbers of expression cassettes with restriction enzymes *Pac*I and *Asc*I. The restriction enzymes sites (*Pac*I and *Asc*I) for identification of the vector are also indicated. P1–P4, primers used for PCR analysis. LB: left border; RB: right border. Marker, 1-kb DNA ladder.

The first round of *in vivo* Cre/*lox*P-mediated recombination reactions was performed between pDES200 and pOSB103-GUS. The correct clones containing the co-integrative plasmid (pDES200-GUS) were obtained through electroporation of SW106 and heat-shock transformation of *E. coli* DH5α, followed by extraction of the plasmid and complete digestion with the homing endonuclease *I-Sce*I to form a linear retrieval plasmid. The second GFP cassette of pOSB102-GFP was amplified with a pair of chimeric primers (Table S3), and transformed to SW106 to prepare the electrocompetent cells. To increase the frequency of subcloning by gap-repair, relatively longer homology arms are recommended; hence the retrieval plasmid previously mentioned could retrieve this DNA fragment conveniently to constitute pDES200-GUS-GFP. By repeating the process described above for additional target genes, gene assembly was carried out to deliver an IFS cassette of pOSB206-IFS to pDES200-GUS-GFP, and then a BADH cassette from pOSB205-BADH. Finally, we obtained a construct pDES200-GUS-GFP-IFS-BADH carrying four different foreign genes, excluding the selectable marker gene HPTII cassette (Figure 4A). Restriction analyses of a series of recombinant constructs containing various numbers of genes are described in Figure 4C.

Another marker-free multigene transformation vector was constructed to further verify the feasibility of the method for linking multiple transgenes. Vectors pDES100 and pOSB103-GUS were co-integrated by *in vivo* Cre/*lox*P-mediated recombination, producing pDES100-GUS. Then, through gene retrieval, the BAR cassette of pOSB104-BAR was joined to pDES100-GUS, resulting in pDES100-GUS-BAR. After another round of recombination with pOSB211, the desired recombinants were selected as pDES100-GUS-BAR-HPT-free (Figure 4B). Furthermore, the skeleton of the intermediate vector, containing an ampicillin-resistance gene for bacterial selection and the ColE1 origin of replication, did not need to be removed, and can be used for plasmid rescue procedures after transformation. We also tested the stability of the co-integrative plasmid of pDES100-GUS-BAR-HPT-free containing two replicons in *E. coli* and *Agrobacterium* using restriction enzymes to confirm that there were no aberrations in size. If the backbone of pDonor with *lox*P was excised, it was necessary to add another *lox*P, as a selectable marker gene should be placed between two *lox*P sites in direct orientation.

### Plant transformation and expression analysis

First we respectively test the effectiveness of each expression cassette. The constitutive overexpression vectors, containing *gfp* gene introduced by gateway recombination, were individually introduced into onion epidermal cells by particle bombardment. GFP was detectable, confirming the activity of each expression cassette on the satellite vector (Figure S2). Moreover, leaf epidermal protoplasts of tobacco transformed with plasmid pOSB108-TP-GFP for chloroplast-targeted were isolated as shown Figure S3.

**Figure 5:**
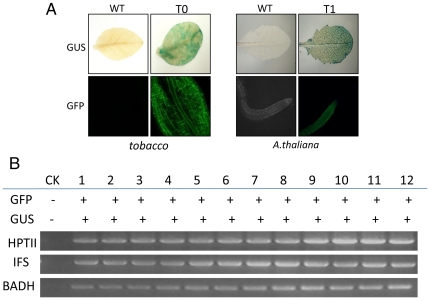
Expression analysis of transgenic plants. (A) Leaves of transgenic plants were taken for *in situ* histochemical staining of GUS activity and roots were imaged by fluorescent confocal microscopy. Only one of the randomly chosen transgenic plants is shown. WT, wild-type; T0, T0 generation of transgenic tobacco; T1, T1 generation of transgenic *A.thaliana*. (B) Expression analysis of the different transcription units of transgenic *A.thaliana*. Confirmation of transgene expression of *gfp* gene and *gus* gene activity is symbolized by +, and lack of transgene activity is symbolized by −. Reverse transcriptase-polymerase chain reaction (RT-PCR) analysis of all gene cassettes (HPTII, IFS, BADH) on the T-DNA from the leaves of 12 transgenic *A. thaliana*.

To evaluate the feasibility of this multigene transformation system, the construct pDES200-GUS-GFP-IFS-BADH was introduced into tobacco and *Arabidopsis. thaliana* by *Agrobacterium*-mediated transformation. Genomic PCR on independent transformants was performed, and 15 independent lines of T0 tobacco transformants were obtained. Because *mgfp5* is not the most highly-fluorescent GFP mutant, and would be masked by the high chlorophyll autofluorescence of leaf cells, so we chose roots to enable easy visualization of the GFP. All of the transgenic plants showed GFP fluorescence and constitutive *in situ* GUS expression (Figure 5A), despite differences in the expression levels. To check for the expression of other genes, reverse transcriptase-polymerase chain reaction (RT-PCR) analysis of transgenic plants was carried out for each expression unit on the T-DNA. The predicted cDNA fragments of all transgenes, that were linked in the same T-DNA region, could be amplified in all tested samples.

After screening on agar-solidified media containing Murashige and Skoog salts (MS agar) with hygromycin (30 mg/L), 26 putative *A. thaliana* T1 transgenic plants were initially generated. Apart from two transgenic T1 lines exhibited poor fertility, 24 independent T2 transgenic *A. thaliana* lines were performed for further gene expression analysis with RT-PCR. Similar results were obtained as with tobacco, we found that all transgenes linked on the T-DNA were coexpressed. (Figure 5B).

**Figure 6:**
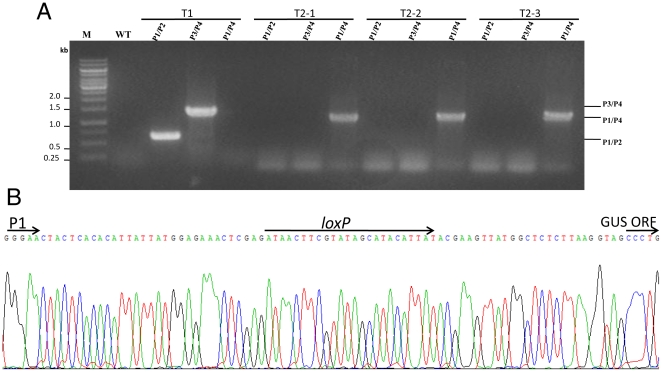
Identification of Cre/*lox*P-mediated DNA recombination event. (A) PCR analysis of genomic DNA prepared from uninduced T1 plant and recombinant T2 transgenic plants (lines1, 2 and 3) using primers P1/P2, P3/P4, and P1/P4. Primer pairs used for each PCR reaction are indicated on the top of each lane. The expected PCR products from different combinations of primer pairs are indicated on the right. No amplification was observed with the genomic DNA of wild-type plants (WT) using any of the three primer pairs. M, 1-kb DNA ladder. (B) Sequence confirmation of marker gene excision in the genome of transgenic T2 lines. Part of the sequence at excision site is exactly as predicted, indicating that the excision occurred and all DNA sequences, including *hpt*II gene, between two *lox*P sites were excised, with only one intact *lox*P site left.

To verify the capability for creating multigene transgenic plant of conditional elimination of selection markers, the construct pDES100-GUS-BAR-HPT-free was also introduced into *A. thaliana* lines by *Agrobacterium*-mediated transformation. Sixteen *A. thaliana* transgenic lines (T1) were obtained and transferred onto the inductive medium. After *β*-estradiol-induction (5 µm) for two weeks, all independent lines were then transferred to soil, and T2 seeds were obtained as a pool. Then we carried out PCR analysis with primer pairs specific for the excised sequences and flanking nonexcised sequences to verify the recombination events. The P1–P2 (612 bp) and P3–P4 (1341 bp) fragments will be amplified respectively in non-recombinant T-DNA, whereas P1–P4 (1184 bp) fragments can be detected in recombinant T-DNA (Table S5). Only those lines with unique P1–P4 fragment could be considered as complete DNA excision. Among the tested 100 lines randomly selected from the pool, 13 lines show complete marker excision (Figure 6A). The PCR products of P1–P4 fragments amplified from three marker-free transgenic plants (T2) were sequenced and all these sequencing results were identical (Figure 6B), indicating that the DNA fragment containing marker gene between two *lox*P sites was removed precisely.

In conclusion, these results indicated that all heterologous genes on the T-DNA could be efficiently delivered into the plant genome as a whole, and could be successfully coexpressed.

## Discussion

As already described, various gene manipulation methods have been created and widely used by means of site-specific recombination and homologous recombination. Cre has long been recognized as the best site-specific recombinase for DNA engineering, allowing the construction of large segments of transgenic DNA [11], [15]. The initial procedure is similar to a univector plasmid-fusion system [26], the Creator cloning system (Clontech), or Echo cloning system (Invitrogen) in terms of the difference between *in vivo* and *in vitro*. MultiSite Gateway [12] and MultiRound Gateway [13] were produced based on the widely-used Gateway technology to stack multigene cassettes. We have integrated three recombinational cloning methods, namely Gateway, Cre/*lox*P and Recombineering to present a versatile ready-to-use plant multigene genetic transformation platform: RMDAP. The Gateway system, has been adopted as a popular choice for generating various constructs because it allows the gene of interest to be easily introduced into specifically designed plasmids without traditional “cut and paste” steps. And the Cre/*lox*P and Recombineering were used to assembly the gene fragments *in vivo*. Phage-based *E. coli* homologous recombination systems, known as recombineering, have recently been developed that now make it possible to subclone or modify DNA cloned into plasmids without the need for restriction enzymes and ligases [27], [28]. Linear DNAs, either double-stranded (ds, usually in the form of PCR products), or single-stranded (ss) synthetic oligonucleotides, are introduced by electroporation and provide homologous substrates (i.e., targeting constructs) to subclone the DNA from BACs via gap repair [29]. Hence, recombineering is a process of small targeting constructs (e.g., pBluescript or pBR322) retrieving large fragments (BACs or PACs). Our approach is more like “reverse recombineering”: a large linear transformation vector retrieves a small gene cassette or DNA fragment.

As noted in previous research, an ideal binary vector system should include the following features: an easy route for cloning the gene of interest under control of a wide variety of promoter and terminator sequences, the ability to simultaneously express several target genes, and a wide choice of selection markers and reporter genes [16]. Although pAUX/SAT plasmids have been developed into a successful vector family for various applications, the initial design of vectors has limited its loading capacity. Moreover, the same pAUX/SAT plasmid cannot be reused directly, and the relatively large insert size (e.g. the DNA fragment for marker-free) ligated to pPZP-RCS is also a limiting factor for applications. RMDAP also has a family of satellite plasmids for different purposes in plant molecular biology, such as overexpresstion and RNAi. The in-built flexibility of the original vectors has greatly increased the expansibility and applicability of the system. Especially, construction of marker-free, multigene transformation vector is an attractive aspect for scientists. In the auto-excision strategy, the recombinase gene and the selectable marker were placed on the same vector between the recombination sites. The marker-free system has also previously proved feasible through activation of recombinase, which can be induced by chemicals (estrogen- and chloroacetanilide- induction) [22], [23]. In particular, tissue-specific promoters, such as germline-specific promoters, can be used to make transgenic plants become genetically programmed to lose the selectable marker when its presence is no longer required [30]. Thus the versatile vector of the marker-free system, allowing us to replace the promoter through Gateway technology, greatly increases its applicability in these areas, as long as there is an efficient specific promoter.

Constructing a highly efficient and easy-to-use vector system is the goal for plant gene expression analysis. It is worth emphasizing that the recombination systems of Cre/*lox*P and Gateway technology have proved to be very efficient and reliable for cloning, and allow easy manipulation of fusion constructs [19], [31]. Recombineering is also an efficient method for *in vivo* genetic engineering for manipulating BAC DNA in *E. coli* [28]. In addition, longer homologies have been used to further improve the targeting efficiency [28]. Our multigene transformation platform, RMDAP, has incorporated these three systems into an easy-to-use method. Moreover, versatile, functionally validated, accessible satellite plasmids greatly simplify the process of vector construction. In short, the system just only requires some simple DNA manipulations in a few days, from a single gene determined to a complete multigene vector. Moreover, if the number of transgenes is relatively small, rare restriction enzymes, including restriction enzymes of recognition sequences of eight bases, could also be used as pAUX/SAT family vectors [16]. We are considering constructing several auxiliary plasmids containing frequently used expression cassettes corresponding to the rare-cutting enzyme sites *I-Sce*I, *I-Ppo*I, *Asc*I, and *Pac*I. Accordingly, this could greatly simplify experimental operations and ensure the basic requirements for crop improvement.

Notably, when carrying out recombineering, the size of DNA fragment should be relatively small owing to the fidelity of the taq enzyme. Therefore, before the experiment, detailed planning should be undertaken; the satellite RNAi and marker-free vectors are preferably assembled through Cre/*lox*P-mediated recombination because of the high efficiency of the *cre* gene *in vivo* [31]. Moreover, because recombineering is a homologous recombination-based method of genetic engineering, there will inevitably be some duplication of sequences with an increase in fragments. Therefore, it is necessary to develop different types of expression cassettes to minimize repeats as much as possible. One aspect that should be considered again here is that the strategy of Lin [11] is an important complement to this method , owing to design of original vectors and because it employs a site-specific recombination system of Cre/*lox*P, which could to some extent avoid errors caused by homologous recombination. Because the pCAMBIA vector a high-copy plasmid, it can only bear relatively smaller fragments, we are also considering constructing a single-copy, high-capacity plant transformation vector of BIBAC and TAC [32], [33] to increase the capacity of recipient vectors.

## Materials and Methods

### Vector construction and bacterial strain

Standard molecular biology procedures and DNA manipulations [34] were performed to make all the constructs. Details of vector construction are available in the Text S1 [35], [36], [37]. If necessary, the vector backbone was dephosphorylated by Alkaline Phosphatase (Promega) prior to ligation. The sequences of primers used are listed in Table S1. All vectors containing the ccdB gene were propagated in the *E. coli* strain DB3.1, which contains the *gyrA462* mutation that enables propagation of Gateway vectors encoding the *ccdB* gene [35]. SW106 cells were maintained at 32°C and used as the host for DNA assembly and integration and DH5α cells were employed for other transformations.

These vectors can be used in a wide range of functional genomics projects in plants and will be distributed to the research community for noncommercial research purposes upon request.

### Subcloning of target genes to the entry vectors

The *bar* gene of pCambia3301, the *gfp* gene of pCambia1302, the *badh* gene of *Atriplex hortensis*, and the *ifs* gene of *Medicago* were amplified with the primers listed in Table S2, and ligated to pDonor 221 by the BP clonase or pCR®8/GW/TOPO® vector according to the manufacturer's instructions (Invitrogen), resulting in pENTR-bar, pENTR-gfp, pENTR-badh, and pENTR-ifs, respectively. The pENTR-gus was directly purchased from Invitrogen.

Due to the design of the experiment, the pENTR vector was used to transfer DNA fragments to the destination modular vectors using LR clonase according to the manufacturer's instructions (Invitrogen). Finally, we obtained the satellite vectors pOSB102-GFP, pOSB103-GUS, pOSB104-BAR, pOSB205-BADH, and pOSB206-IFS.

### Multiple rounds of recombination reactions

For Cre/*lox*P-mediated plasmid co-integration, plasmids pOSB and pDES were co-electroporated into electro-competent cells of *E. coli* strain SW106, in which the *cre* gene had been induced by adding arabinose. Through screening on LB plates with kanamycin (50 mg/L) and ampicillin (50 mg/L), the resulting plasmid population was obtained and introduced into *E. coli* strain DH5α at a very low concentration to obtain a pure clonal population. After another round of screening as described above, co-integrative clones were obtained, isolated (AxyPrep Plasmid Miniprep Kit, Axygen), and digested completely with homing endonuclease (New England BioLabs and Promega) to produce the linear targeting vector.

For recombineering-mediated gene retrieval, chimeric primers were precisely designed according to the flanking sequence of the homing endonuclease site of the linear targeting vector and the second DNA fragment. The fragment was amplified with Phusion High-Fidelity DNA Polymerase (New England BioLabs) and ligated to the pMD18-T simple vector (Takara). The resulting plasmid was transformed to *E. coli* SW106 to grow at 32°C, and then the cultures were transferred to 42°C for a 15-min induction of *Red* recombination functions to prepare the electrocompetent cells. Finally, electroporation of the linear targeting vector (300 ng) was performed using a Bio-Rad electroporator under the following conditions: 1.80 kV, 25 mF capacitance, and 200 Ω resistance. Cells were spread on plates with kanamycin(50 mg/L) to screen for the correct recombinant clones by restriction analysis [29].

### Plant transformation and expression analysis

Plants of Arabidopsis (*A. thaliana* Col-0), *N. tabacum cv. Xanthi* and *Nicotiana benthamiana* were placed in a growth chamber (25°C under a 16-h-light/8-h-dark cycle). The constitutive ectopic overexpression vectors were used for transient expression either of onion epidermis via a gene gun (Bio-Rad) or of *N. benthamiana* leaves via Agro-infiltration [38].

The *A. tumefaciens* strain *Eha105* was used to transform tobacco via the leaf disc system [39] and *A. thaliana* Columbia-0 via the floral dipping method [40]. Six- to 8-week-old *N. tabacum cv. Xanthi* plants were transformed and explants were selected on MS medium containing 20mg/L hygromycin (Amersham Biosciences). The successful insertion of the exogenous genes was confirmed by PCR with genomic DNA isolated from leaves of independent transformants as templates. Transformed *A. thaliana* T1 seeds were selected on germination medium plates supplemented with 30 mg/L hygromycin, and T1 seedlings were transferred on an inductive medium containing 5 µM of 17-β-estradiol for 2 weeks.

Plant tissues were viewed directly for fluorescence analysis under a Nikon ECLIPSE TE2000-E inverted fluorescence microscope equipped with a Nikon D-ECLIPSE C1 spectral confocal laser scanning system. The *in situ* histochemical GUS assays were conducted using 5-bromo-4-chloro-3-indolyl glucuronide (Amersham Biosciences) as substrate [41]. The leaf tissues were cleared of chlorophyll by applying 90%, 80%, and 70% ethanol solutions in three consecutive steps, and photographed using a stereomicroscope.

Total RNA was extracted from leaf tissue with Trizol reagent (Invitrogen) according to the manufacturer's recommendations and digested with DNase I (New England BioLabs) to remove genomic DNA. The first strand of cDNA was synthesized using 5 µg of total RNA as template with the Reverse Transcription System (Promega) in a 20 µl reaction volume. Primers for each gene and size of fragment are shown in Table S4.

## Supporting Information

Figure S1 **Structural features of the pOSB208 that allow recombination of the protein of interest for in-frame fusions to the chloroplast transit peptide (TP).**
(DOC)

Figure S2 **Transient expression of constitutive ectopic overexpression vectors by particle bombardment into onion epidermis.**
(DOC)

Figure S3 **Tobacco protoplasts were transformed with plasmids pOSB208-TP-GFP of transit peptide to the N terminus of** ***gfp*** **.**
(DOC)

Table S1 **The primers for vector construction of system.**
(DOC)

Table S2 **The primers for construction of pDonor vector.**
(DOC)

Table S3 **The primers for gene stacking.**
(DOC)

Table S4 **The primers for RT-PCR and expected band size.**
(DOC)

Table S5 **The primers for identifying the recombination events.**
(DOC)

Text S1 **Details of vector construction.**
(DOC)
