# Supplementary material for: RMDAP: A Versatile, Ready-To-Use Toolbox for Multigene Genetic Transformation
Source: PLoS One. 2011 May 13;6(5):e19883. doi: 10.1371/journal.pone.0019883 (PMC3094388; doi:10.1371/journal.pone.0019883)
Supplement: Figure S1 — Structural features of the pOSB208 that allow recombination of the protein of interest for in-frame fusions to the chloroplast transit peptide (TP). (DOC) [file pone.0019883.s001.doc]

**
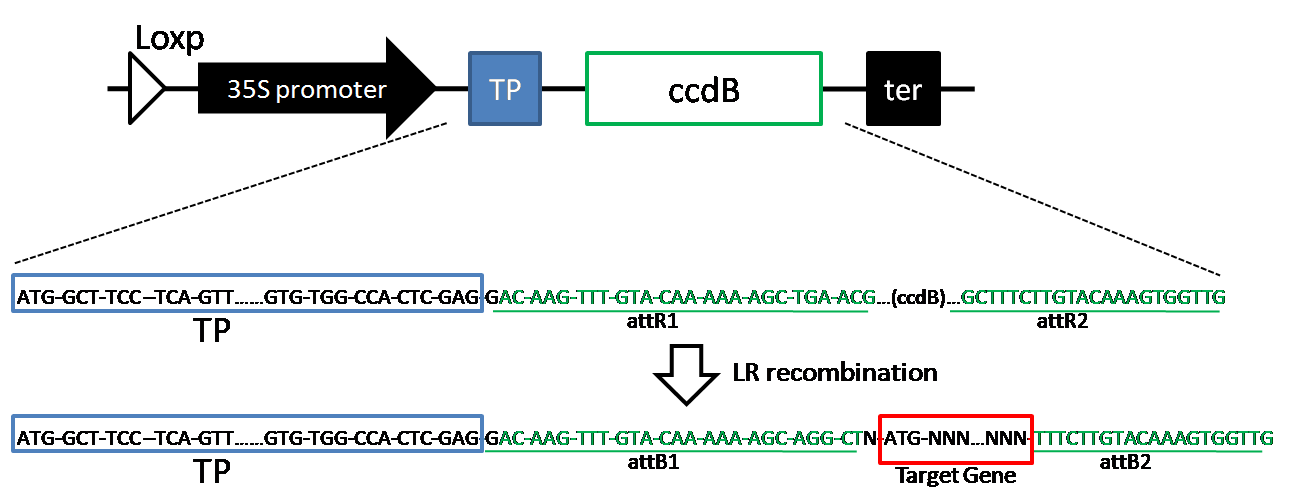
**

**Figure S1**：

Structural features of the pOSB208 that allow recombination of the protein of interest for in-frame fusions to the chloroplast transit peptide (TP). The CaMV35S promoter is set up on the upstream of the transit peptide to drive the expression of target gene. The recombination events between the *att*L and *att*R sites that generate *att*B sites occur, so the target gene could in-frame fusion to transit peptide. The sequences of the target gene fusion junction, after the attL/attR recombination, are shown. Transit peptide (TP) sequences are indicated by blue boxes, target gene sequences are indicated by red boxes, and recombination sites are indicated by green font.
