# Supplementary material for: RMDAP: A Versatile, Ready-To-Use Toolbox for Multigene Genetic Transformation
Source: PLoS One. 2011 May 13;6(5):e19883. doi: 10.1371/journal.pone.0019883 (PMC3094388; doi:10.1371/journal.pone.0019883)
Supplement: Figure S2 — Transient expression of constitutive ectopic overexpression vectors by particle bombardment into onion epidermis. (DOC) [file pone.0019883.s002.doc]

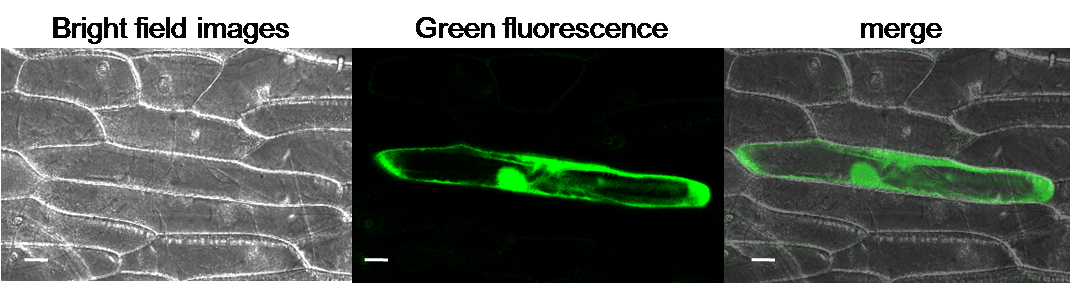


**Figure S2**：

Transient expression of constitutive ectopic overexpression vectors by particle bombardment into onion epidermis. Similar results were obtained, and only the image of pOSB104-GFP was presented. Bright-field images, Green fluorescence signals, and a merge are shown. Bars=50 μm.
