# Supplementary material for: RMDAP: A Versatile, Ready-To-Use Toolbox for Multigene Genetic Transformation
Source: PLoS One. 2011 May 13;6(5):e19883. doi: 10.1371/journal.pone.0019883 (PMC3094388; doi:10.1371/journal.pone.0019883)
Supplement: Figure S3 — Tobacco protoplasts were transformed with plasmids pOSB208-TP-GFP of transit peptide to the N terminus of gfp . (DOC) [file pone.0019883.s003.doc]

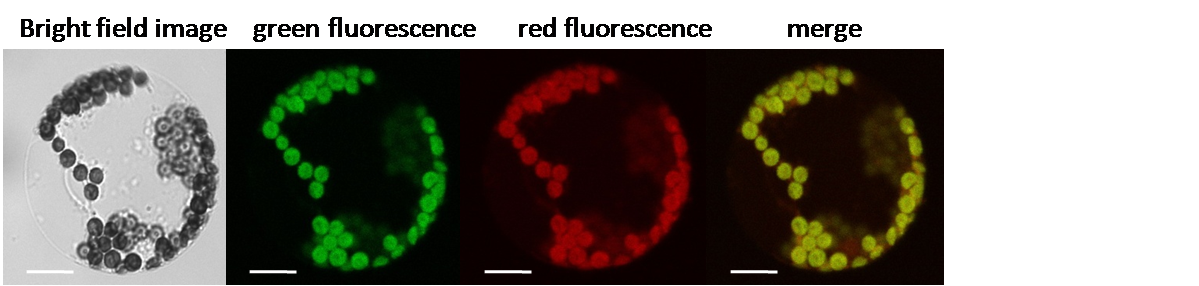


**Figure S3:**

Tobacco protoplasts were transformed with plasmids pOSB208-TP-GFP of transit peptide to the N terminus of *gfp*. Bright-field images, Green fluorescence signals, chlorophyll red autofluorescence, and a merge of green and red signals are shown. Bars=10 μm.
