# Supplementary material for: RMDAP: A Versatile, Ready-To-Use Toolbox for Multigene Genetic Transformation
Source: PLoS One. 2011 May 13;6(5):e19883. doi: 10.1371/journal.pone.0019883 (PMC3094388; doi:10.1371/journal.pone.0019883)
Supplement: Table S1 — The primers for vector construction of system. (DOC) [file pone.0019883.s004.doc]

**Table S1:** The primers for vector construction of system. Restriction sites are in red.

| NO. | Sequence (5’→3’) | name |
| --- | --- | --- |
| P1 | CTCTCTTAAGGTAGCCCTGCAGGGGCGCGCCTAATACGACTCACTATAGGGC | *F-*pOSB1-1 |
| P2 | *ATAACTTCGTATAGCATACATTAT*ACGAAGTTATGGCTCTCTTAAGGTAGCCCTGC | *F-*pOSB1-2 |
| P3 | ATTACCCTGTTATCCCTAGTTTAAACTTAATTAACCCTCACTAAAGGG | *R-* pOSB1-1 |
| P4 | TTAGGGATAACAGGGTAATGTTTAAACTTAATTAATACGACTCACTATAGGGCG | *F-*pOSB2-1 |
| P5 | *ATAACTTCGTATAGCATACATTAT*ACGAAGTTATTAGGGATAACAGGGTAATG | *F-*pOSB2-2 |
| P6 | GCTACCTTAAGAGAGCCTGCAGGCGCGCCAATTAACCCTCACTAAAGGG | *R-* pOSB2-1 |
| P7 | CTCGAGGACAAGTTTGTACAAAAAAGC | *F-*attR1-ccdB-attR2 |
| P8 | AGATCTAGACCACTTTGTACAAG | *R-* attR1-ccdB-attR2 |
| P9 | TTAATTAACATGGAGTCAAAGATTC | *F-*p*35S* |
| P10 | CTCGAGAGTCCCCCGTGTTCTC | *R-*p*35S* |
| P11 | TCTAGA GTCCGCAAAAATCACCAG | *F-*t*35S* |
| P12 | GGCGCGCC CTGGATTTTGGTTTTAGG | *R-*t*35S* |
| P13 | TTAATTAAGATCATGAGCGGAGAATTAAGG | *F-*p*NOS* |
| P14 | CTCGAGAGATCCGGTGCAGATTATTTG | *R-*p*NOS* |
| P15 | TCTAGA GATCGTTCAAACATTTGGC | *F-*t*NOS* |
| P16 | GGCGCGCC CGATCTAGTAACATAG | *R-*t*NOS* |
| P17 | GTTAATTAACCGGGCTGAAAGCGACG | *F-*p*OCS* |
| P18 | CTCGAGATCTGAGTCCGG | *R-*p*OCS* |
| P19 | CTCTAGAACTAGTCCCTAGAGTCC | *F-*t*OCS* |
| P20 | GGCGCGCCTCCTGCTGAGCCTCGAC | *R-*t*OCS* |
| P21 | GTTAATTAATGAGATTTTTCAAATCAG | *F-*p*MAS* |
| P22 | CTCGAGTCGATTTGGTGTATCGAG | *R-*p*MAS* |
| P23 | CTCTAGATTGGACTCCCATGTTGG | *F-*t*MAS* |
| P24 | GGCGCGCCGATAATTTATTTGAAAATTC | *R-*t*MAS* |
| P25 | GTTTAAACGGGATCTTCTGCAAGCATC | *F-*p*ENTCUP2* |
| P26 | CTCGAGTCCGGTGGGTTTTGAGGTG | *R-*p*ENTCUP2* |
| P27 | TTAATTAACAGTGCAGCGTGACCCGG | *F-*p*UBI* |
| P28 | GGTACCAAGTAACACCAAACAACAGGG | *R-*p*UBI* |
| P29 | TTAATTAA ACGCGTATGGCTTCCTCAGTTCTTTC | *F-*TP1 |
| P30 | CTCGAGTGGCCACACCTGCATG | *R-*TP1 |
| P31 | ACGCGTAGTCCCCCGTGTTCTC | *R-*p*35S2* |
| P32 | GACTAGTATAGTTTAAACTGAAGGCGGG | *F-*G10-90F |
| P33 | GGAATTCGGATCCCAGCGTGTCCTC | *R-*G10-90R |
| P34 | TCGCGATTATCTTCTATATCTTC | *F-*CRE |
| P35 | CCTCGAGTAGTAACATAGATGACACCGCGCGC | *R-*CRE |
| P36 | ACGCGTCC TCGCGAGTCCGCAAAAATCACCAG | *F-*t*35S2* |
| P37 | CTCGAGCTGGATTTTGGTTTTAGG | *R-*t*35S2* |
| P38 | GACGCGTCGAGCTTTCGCAGATCC | *F-*hpt |
| P39 | TCGCGACTATTTCTTTGCCCTCGG | *R-*hpt |
| P40 | GACGCGTATGATTGAACAAGATGG | *F-*Kan |
| P41 | TCGCGATCAGAAGAACTCGTCAAGAAG | *R-*Kan |
| P42 | CTCTAGAGTCGACTCTAGCCTCG | *F-*CreT |
| P43 | GGCGCGCCTAGTAACATAGATGACACC | *R-*CreT |
| P44 | GGGGACAAGTTTGTACAAAAAAGCAGGCT GGCGTTGAGCCTTTTTCTAC | *F-*In5-2 |
| P45 | GGGGACCACTTTGTACAAGAAAGCTGGGT GTTTCCTGCTACTCGTTGG | *R -*In5-2 |
| P46 | CTCGAG ATAACTTCGTATAGCATACATTATACGAAGTTAT AACAGGGACACCA GGATTTATTTATTCTGCG | *F-DES100* |
| P47 | AAGCTTAATTAAGTTTAAACATTACCCTGTTATCCCTACAACCACTTTGTACAAGAAAGC | *R-DES100* |
| P48 | GAATTCATAACTTCGTATAGCATAC | *F-DES200* |
| P49 | GGGTAACCTTAATTAAGTTTAAACATTACCCTG | *R-DES300* |
