# Supplementary material for: RMDAP: A Versatile, Ready-To-Use Toolbox for Multigene Genetic Transformation
Source: PLoS One. 2011 May 13;6(5):e19883. doi: 10.1371/journal.pone.0019883 (PMC3094388; doi:10.1371/journal.pone.0019883)
Supplement: Table S2 — The primers for construction of pDonor vector. (DOC) [file pone.0019883.s005.doc]

**Table S2:** The primers for construction of pDonor vector

| Name | Sequence (5’→3’) |
| --- | --- |
| *F-bar* | GGGGACAAGTTTGTACAAAAAAGCAGGCT ATGAGCCCAGAACGACG |
| *R-bar* | GGGGACCACTTTGTACAAGAAAGCTGGGT TCAGATCTCGGTGACGGGC |
| *F-mgfp* | GGGGACAAGTTTGTACAAAAAAGCAGGCT ATGGTAGATCTGACTAGTAAAGG |
| *R-mgfp* | GGGGACCACTTTGTACAAGAAAGCTGGGT TCACACGCTAGCTTTGTATAGTTCATC |
| *F-badh* | CTCGAGATGGCGTTCCCAATTCCTG |
| *R-badh* | TCTAGATCAAGGAGACTTGTACCATC |
| *F-ifs* | CTCGAGATGTTGGTGGAACTTGCAG |
| *R- ifs* | TCTAGATTAGGAGGAAAGAAGTTTATC |
