# Supplementary material for: RMDAP: A Versatile, Ready-To-Use Toolbox for Multigene Genetic Transformation
Source: PLoS One. 2011 May 13;6(5):e19883. doi: 10.1371/journal.pone.0019883 (PMC3094388; doi:10.1371/journal.pone.0019883)
Supplement: Table S3 — The primers for gene stacking. (DOC) [file pone.0019883.s006.doc]

**Table S3:** The primers for gene stacking

| Name | Sequence (5’→3’) |
| --- | --- |
| F-35S-ORF | GACTCCCTTAATTCTCCGCTCATGATCTTAATTAAGTTTAAACTAGGGATAA TAATTCGGGGGATCTGGATTTTAG |
| R-35S-ORF | TAAAACGACGGCCAGTGCCAAGCTTAATTAAGTTTAAACATTACCCTG  TTAT CATGGAGTCAAAGATTCAAATAG |
| F-mas-ORF | GCTTTTCTTATGAATTTTCAAATAAATTATCGGCGCGCCTGCAGGCTCT  CTTAA TGAGATTTTT CAAATCAGTG |
| R-mas-ORF | CGGTGTCATCTATGTTACTAGATCGGGCGCGCCCCTGCAGGGCTACCTTAA  GATCTGATAATTTATTTGAAAATTC |
| F-ocs-ORF | GACTCCCTTAATTCTCCGCTCATGATCTTAATTAAGTTTAAACTAGGGATAA TCCTGCTGAGCCTCGACATGTTG |
| R-ocs-ORF | AAACGACGGCCAGTGCCAAGCTTAATTAAGTTTAAACATTACCCTGTTAT  CTGAAAGCGA CGTTGGATG |
