# Supplementary material for: RMDAP: A Versatile, Ready-To-Use Toolbox for Multigene Genetic Transformation
Source: PLoS One. 2011 May 13;6(5):e19883. doi: 10.1371/journal.pone.0019883 (PMC3094388; doi:10.1371/journal.pone.0019883)
Supplement: Table S4 — The primers for RT-PCR and expected band size. (DOC) [file pone.0019883.s007.doc]

**Table S4:** The primers for RT-PCR and expected band size.

| Name | Sequence (5’→3’) | Size of fragment |
| --- | --- | --- |
| *hptII* | ATGAAAAAGCCTGAACTC | 1026bp |
|  | CTATTTCTTTGCCCTCGG |  |
| *ifs* | TGAACGACTTGCTCAACGCC | 830bp |
|  | TTCCAATGGCTTTACCC |  |
| *badh* | TTCTGGTGCTCATCGTGC | 510bp |
|  | CTCCCAGTAAATGCTACCTTG |  |
| *gus* | CTGTTGACTGGCAGGTGGTG | 919bp |
|  | ACGCCGTATTCGGTGATG |  |
| *gfp* | ATGGTAGATCTGACTAG | 756bp |
|  | TCACACGTGGTGGTGGTGGTG |  |
