# Supplementary material for: RMDAP: A Versatile, Ready-To-Use Toolbox for Multigene Genetic Transformation
Source: PLoS One. 2011 May 13;6(5):e19883. doi: 10.1371/journal.pone.0019883 (PMC3094388; doi:10.1371/journal.pone.0019883)
Supplement: Table S5 — The primers for identifying the recombination events. (DOC) [file pone.0019883.s008.doc]

**Table S5:** The primers for identifying the recombination events

| P1 | CTTAATAACACATTGCGGACG |
| --- | --- |
| P2 | GGATCCCAGCGTGTCCTC |
| P3 | CTCACATGTTCTTTCCTGCG |
| P4 | TGGAGTATTGCCAACGAAC |
