# Supplementary material for: RMDAP: A Versatile, Ready-To-Use Toolbox for Multigene Genetic Transformation
Source: PLoS One. 2011 May 13;6(5):e19883. doi: 10.1371/journal.pone.0019883 (PMC3094388; doi:10.1371/journal.pone.0019883)
Supplement: Text S1 — Details of vector construction. (DOC) [file pone.0019883.s009.doc]

**Text S1:**

All vectors containing the ccdB gene were propagated in the *E. coli* strain DB3.1, which contains the *gyrA462* mutation that enables propagation of Gateway vectors encoding the *ccdB* gene (Hartley *et al.*, 2000). SW106 cells were maintained at 32°C and used as a host for DNA assembly and integration. DH5α cells were employed for other transformations. All of the primers used for vector construction are presented in table S1.

The backbone of two original entry vectors, pOSB100 and pOSB200, was constructed based on the pMD18-T simple vector (Takara), a high-copy number cloning vector that can facilitate isolation of a large amount of plasmid DNA, and contains the ampicillin resistance gene. The fragment, encompassing a *loxP* site, a multicloning site (MCS) from pBluescript II SK (Stratagene), and two restriction enzyme sets arrayed in order as described in Figure 1, was obtained by overlap PCR and ligated to pMD18-Tsimple. The clone containing the right direction of the fragment was selected. The forward primers P1 and P2, and reverse primer P3 were employed for pOSB100, and the forward primers P4 and P5, and reverse primer P6 were used for pOSB200. Restriction enzyme set 1 contains *Asc*I, *Sbf*I, and *I-Ppo*I.， and restriction enzyme set 2 contains *Pac*I, *Pme*I, and *I-Sce*I. These two sets have opposite orientation.

All of the vectors described below, in addition to special illustrations, present the construction process of the pOSB2xx vector. Satellite vector pOSB1xx was constructed by introducing the *Pac*I-*Asc*I fragment of pOSB2xx. To keep the size of the modular vector to a minimum for convenient *in vitro* manipulation, the 1.4-kb gateway cassette containing the negative selection marker ccdB (chloromycetin excluded) was amplified by primers P7 and P8 from phellsgate8 as a *Xho*I-*Xba*I fragment and then subcloned into pOSB200, resulting in pOSB201. Six constitutive promoters and four terminators were used to construct the over-expression vector series: cauliflower mosaic virus 35S promoter (p*35S*) (primers P9 and P10) and terminator (t*35S*) (primers P11 and P12) from pCambia1302, nopaline synthase gene promoter (p*NOS*) (primers P13 and P14) and nopaline synthase gene terminator (t*NOS*) (primers P15 and P16) from pBI121, octopine synthase promoter (p*OCS*) (primers P17 and P18) and octopine synthase terminator (t*OCS*) (primers P19 and P20) from pPZP-RCS2-BAR, manopine synthase promoter (p*MAS*) (primers P21 and P22) and manopine synthase terminator (t*MAS*) (primers P23 and P24) from pEarleyGate100, and maize ubiquitous promoter (p*UBI*) (primers P27 and P28) from pCXUN. These were all cloned as the *Pac*I-*Xho*I fragment for the promoter or the *Xba*I-*Asc*I fragment for the terminator, and inserted into the same sites of pOSB201 to produce a series of modular vectors. Tobacco cryptic promoter (p*ENTCUP2*) (primers P25 and P26) from pORE T3 was cloned as the *Pme*I-*Xho*I fragment and introduced to the same sites of pOSB201 to produce pOSB206.

The signal peptide of the tobacco gene ribulose 1,5-bisphosphate carboxylase small subunit was amplified with primers P29 and P30 to add a *Pac*I and an *Mlu*I to the 5’ termini and *Xho*I to the 3’ termini. The clone of the fragment of cauliflower mosaic virus 35S promoter (p*35S*), with primers P9 and P31 as *Pac*I and *Mlu*I fragments, was inserted into the same sites of 5’ termini of the signal peptide. The resulting segment was digested with *Pac*I and *Xho*I to yield p*35S*+TP and introduced into pOSB204, producing pOSB208.

The phellsgate2 and phellsgate8 vectors were derived from pH/KANNIBAL (Helliwell *et al.*, 2002), and the RNAi cassette was subcloned as a *Not*I fragment containing the hpRNA cassettes into pOSB100 and pOSB200, producing BP-mediated pOSB109 and pOSB209, and LR-mediated pOSB110 and pOSB210.

The estrogen-induced silencing element from pX7-GFP(Guo *et al.*, 2003) was divided into four parts, which were introduced into the module vector successively. First, a synthetic 84-bp DNA segment of VXE (from ATG to *Mlu*I site) was obtain with *Pac*I-*Spe*I-*EcoR*I sites on the 5’ terminal and *Mlu*I-*Nru*I-*Xho*I-*Asc*I sites on the 3’ terminal in that order. Second, the G10-90 promoter was amplified with primers P31 and P32 to be digested with *Spe*I and *EcoR*I and inserted into the same sites of the synthetic segment. Third, Vector pX7-GFP was digested with *Mlu*I and *Nru*I, and the resulting fragment was also inserted into the synthetic segment. Fourth, P33 and P34 primers were used to amplify the posterior region of the CRE ORF as a *Nru*I-*Xho*I fragment into the synthetic DNA segment. Finally, the resulting fragment was digested with *Pac*I and *Asc*I and inserted into the parental entry vector pOSB200, producing pOSB211.

The 35S terminator was amplified from p*Cambia1302* with primers P36 and P37 introducing *Mlu*I-*Nru*I to the 5’ termini and *Xho*I to the 3’ termini. The *hptII* gene from p*Cambia*1300 with primers P38 and P39, and *Kan* gene of p*BI*121 with primers P40 and P41, were amplified as a fragment inserted into the *Mlu*I and *Nru*I sites of the 35S terminator, producing *HptII*+35S ter or *Kan*+35S ter. This fragment was digested with *Mlu*I and *Xho*I and cloned into pOSB204, resulting in pOSB-HptII and pOSB-Kan. The CRE+ter (from the *Nru*I site of the *cre* gene to the terminator) fragment was amplified from pX7-GFP with primers P42 and P43, and cloned into the *Xba*I and *Asc*I sites of pOSB-HptII and pOSB-Kan, producing pOSB-213 and pOSB-214.

The chloroacetanilide-induced promoter (*In5-2*) (Yuan *et al.*, 2004) was amplified with primers P44 and P45 and inserted into pDonor221, producing pEntry-In5-2. Through the LR clonase, promoter In5-2 was cloned into pOSB211, yielding pOSB212.

The backbone of all stable expression vectors is derived from p*CAMBIA* (CAMBIA, Center for the Application of Molecular Biology to International Agriculture, Canberra, Australia), an *Agrobacterium tumefaciens* binary vector widely used for transformation in both dicot and monocot plants. The receptor fragment containing loxP and restriction enzyme set 1 was amplified from pHellsgate8 with primers P46 and P47, and introduced into the *Xho*I and *Hind*III sites of pCambia1300, producing pDES100. Primers P48 and P47 were employed to clone the receptor fragment, and inserted into the *EcoR*I and *Hind*III sites of pCambia1300, producing pDES200. The primers P48 and P49 were used to amplify pDES200 as a fragment, and ligated to the *Hind*III and *BstE*II sites of pCambia3301, yielding pDES300.

**Reference:**

Guo HS, Fei JF, Xie Q, Chua NH (2003) A chemical-regulated inducible RNAi system in plants. Plant J 34: 383-392.

Hartley JL, Temple GF, Brasch MA (2000) DNA cloning using in vitro site-specific recombination. Genome Research 10: 1788-1795.

Helliwell CA, Wesley SV, Wielopolska AJ, Waterhouse PM (2002) High-throughput vectors for efficient gene silencing in plants. Functional Plant Biology 29: 1217-1225.

Yuan Y, Liu YJ, Wang T (2004) A new Cre/lox system for deletion of selectable marker gene. Acta Botanica Sinica 46: 862-866.
